# Are atopy and eosinophilic bronchial inflammation associated with relapsing forms of chronic rhinosinusitis with nasal polyps?

**DOI:** 10.1186/s12948-015-0026-8

**Published:** 2015-09-11

**Authors:** Mona-Rita Yacoub, Matteo Trimarchi, George Cremona, Sara Dal Farra, Giuseppe Alvise Ramirez, Valentina Canti, Emanuel Della Torre, Mattia Baldini, Patrizia Pignatti, Mario Bussi, Maria Grazia Sabbadini, Angelo A Manfredi, Giselda Colombo

**Affiliations:** Allergy and Clinical Immunology Unit, IRCCS San Raffaele Hospital, Via Olgettina 60, 20132 Milan, Italy; Vita-Salute San Raffaele University, Milan, Italy; Allergy and Immunology Unit, Fondazione Salvatore Maugeri, IRCCS, Pavia, Italy

**Keywords:** Rhinosinusitis, Nasal polyps, Atopy, Relapse, Eosinophils

## Abstract

**Background:**

The aetiopathogenesis of chronic rhinosinusitis with nasal polyps (CRSwNP) is still unknown. The role of atopy and the concept of united airways in such patients are still a matter of debate. In this pilot study we aimed at evaluating the degree of eosinophilic inflammation and the frequency of atopy in a cohort of CRSwNP patients candidate for Functional Endoscopic Sinus Surgery (FESS) and assessing the association between these factors and relapsing forms of CRSwNP.

**Methods:**

30 patients (18 men, 12 women) with CRSwNP eligible for FESS were evaluated before and after surgery. Preoperative investigation included: history of previous relapse after FESS, clinical and laboratory allergologic assessment, spirometry, methacholine challenge, blood eosinophilia and determination of the fraction of nitric oxide in exhaled air (FeNO). Nasal fibroendoscopy, spirometry and FeNO determination were also assessed prospectively at 3 and 27 months post-FESS.

**Results:**

18/30 subjects were atopic, 6/18 (33 %) were monosensitized, 16/30 (53 %) were asthmatics and 10/30 (33 %) had non steroidalantinflammatory drugs (NSAIDs) hypersensitivity. Twenty-one patients (70 %) were classified as relapsers, 15/18 (83 %) among atopics, 6/12 (50 %) among non atopics (*p* = 0.05). Among patients with NSAIDs hypersensitivity, 9/10 (90 %) were relapsers. The median IgE concentration was 161.5 UI/mL in relapsers and 79 UI/mL in non-relapsers (ns). The mean FeNO decreased after FESS (43.1–26.6 ppb) in 84 % of patients, but this effect disappeared over time (FeNO = 37.7 ppb at 27 months). Higher levels of FeNO pre-FESS were detected in atopics, and in particular in relapsing ones (median 51.1 ppb vs 22.1, ns). Higher levels of FeNO pre-FESS were detected in asthmatic patients, especially in those who relapsed (median: 67 vs 64.85 ppb in non-relapsed patients, ns). The Tiffeneau Index (FEV1/FVC) was significantly lower in asthmatic relapsers than in non relapsers asthmatics (94.7 ± 11.1 versus 105 ± 5.9—*p* = 0.04). Patients with asthma and atopy had a major risk of relapse (*p* = 0.05).

**Conclusion:**

In our pilot study, atopy, severe asthma, bronchial inflammation, NSAIDs hypersensitivity and high level of total IgE are possible useful prognostic factors for the proneness to relapse after FESS. The role of allergy in CRSwNP pathogenesis should consequently be given deeper consideration. Allergen specific immunotherapy, combined with anti-IgE therapy, may have an immunomodulatory effect preventing polyps relapse and need to be investigated.

**Electronic supplementary material:**

The online version of this article (doi:10.1186/s12948-015-0026-8) contains supplementary material, which is available to authorized users.

## Background

Chronic rhinosinusitis (CRS) affects approximately 5–15 % of the general population both in Europe and the USA, ranking this condition second in prevalence among all chronic conditions [1–3]. Only a fraction of CRS patients develop nasal polyps (NP), suggesting that nasal inflammation could follow different pathogenic pathways. The reported prevalence of NP is 2.7 % in population-based studies [4]. NP occur in all races with a slight male preponderance (male to female ratio = 2.2:1) [5] and with an increased frequency in asthmatics. Besides congenital subtypes (e.g. those secondary to cystic fibrosis), most forms of NP have their onset in the early adulthood [6–9]. However, all age groups are involved and the prevalence of the disease grows with age.

Little is known about the pathogenesis of CRS with or without NP (CRSwNP and CRSnNP respectively). As a consequence, most patients are classified as having idiopathic CRS in the daily clinical practice. Indeed chronic inflammation of the mucosa of the nose and paranasal sinuses develops in almost all cases, maybe as the evolution of acute rhinosinusitis (especially in cases of CRSnNP). According to the current pathogenic paradigm, genetic susceptibility [10, 11] and environmental triggers probably concur to the failure of the normal immunological surveillance at the level of the airway mucosa and to the development of aberrant immune responses against unknown allergens/antigens [12]. In particular, chronic bacterial colonization of the respiratory mucosa is thought to promote IgE production through superantigen stimulation [12, 13] and subsequently to enhance the degranulation of mast cells within the polyps [14]. This phenomenon would explain the known discrepancy between the high local expression of IgE in the mucosa of asthmatic and CRSwNP (but not CRSnNP) patients and the poor correlation of this finding with serum IgE concentrations and skin prick test results [15].

The prognosis of CRS is worse in CRSwNP than in CRSnNP, especially if the patient is suffering also from asthma and non steroidalantinflammatory drugs (NSAIDs) hypersensitivity [16, 17]. CRSwNP is in fact characterized by invalidating symptoms, that alter patient quality of life. Furthermore the rate of post-surgical recurrence is high (60 %) in these patients, when compared to patients with CRSnNP. However, affordable markers of disease activity and predictors of relapse are not yet available for the Clinician to date.

We thus aimed at identifying possible associations between a panel of clinical, laboratory and functional markers of atopy and eosinophilic inflammation and the disease course of patients with CRSwNP before and after FESS.

## Methods

### Patients

30 CRSwNP patients, aged 18–75 years and candidate to FESS were consecutively enrolled. The study was approved by the Ethical Committee of the SanRaffaele Hospital (Milan, Italy) and all subjects signed informed consent. CRSwNP was diagnosed by fibroscopy and CT scan without contrast media. Fourteen patients had already undergone FESS in the past. Patients with previous relapses and patients with evidence of relapse at 27 months were all included in the “relapsers’” subgroup. Exclusion criteria were represented by associated congenital diseases (Cystic fibrosis), granulomatous or vasculitic diseases (Sarcoidosis, Granulomatosis with Polyangiitis, Eosinophilic Granulomatosis with Polyangiitis).

Clinical, laboratory and functional assessments included:

- ***Allergy assessment***: *Clinical history*: exposure to environmental agents: occupational agents, pets, smoke, familiarity for allergic diseases, asthma, rhinosinusitis, autoimmune diseases. Past allergic clinical history: rhinocongiuntivitis, rhinosinusitis, asthma, drug hypersensitivity, food allergy. Allergy tests already performed. Extra-allergic clinical history and current medical/surgical treatment were also assessed. All patients underwent *skin prick tests (SPTs)* for common aeroallergens (house dust mites, mould (Alternaria, Cladosporium, Aspergillus), pollens (Hazel, Alder, Birch, Cypress, Grass, Olive, Parietaria, Mugwort and Ragweed), latex and cat and pet dander as previously described [18].
- ***Laboratory assessments***: all patients underwent a complete blood count, erythrosedimentation rate (ESR), C-reactive protein (CRP), protein electrophoresis, antinuclear antibodies, anti-neutrophil cytoplasmic antibody, total and specific IgE for the aeroallergens panel previously described for SPTs, in case of negative in vivo tests. *Blood hypereosinophilia* was defined as eosinophils >500/mm^3^. Patients were classified as *atopic* when SPT and/or specific IgE were positive to at least one common aeroallergen.
- **Lung function test:***Spirometry*: lung function tests were performed using the standard protocols according to the American Thoracic Society and the European Respiratory Society (ATS/ERS) guidelines [19]. Forced expiratory volume in 1 s (FEV1) and vital capacity (VC) were expressed as a percentage of predicted value. Tiffeneau index (TI) is the ratio of FEV1/forced vital capacity (FVC), used in the diagnosis of obstructive airways diseases. Asthmatic patients were receiving a standard long-term control medication for asthma. *Methacholine challenge* was performed to assess aspecific bronchial hyper reactivity and was made according to the ATS/ERS guidelines in patients without a previous asthma diagnosis and with a normal spirometry at baseline.
- **Offline measurement of the fraction of nitric oxide (NO) in exhaled air (FeNO).** This procedure was performed according to the ATS/ERS guidelines [20].

### Timing

Patients were first evaluated 3 months after FESS by performing: (1) Ear, nose and throat (ENT) visit with fibroendoscopy. (2) Allergy visit. (3) Complete blood count. (4) Determination of FeNO in exhaled air. (5) Lung function test. At 27 months patients were re-evaluated through (1) ENT visit with fibroendoscopy. (2) Allergy visit and (3) determination of FeNO in exhaled air.

## Statistics

Data are presented as mean ± standard deviation (SD) or median ± IQR. For group comparisons, we used the Mann–Whitney U test. Categorical variables were compared with the Fisher’s exact test. *P* values less than 0.05 were considered statistically significant. Relative risk for relapse of CRSwNP co-morbidities was also calculated.

## Results

The clinical features of the 30 CRSWNP patients (19 males, 11 females) are shown in Table 1. Eighteen patients were atopic (11 of them newly diagnosed), 16/30 were asthmatics (9 of them newly diagnosed) and 10/30 had NSAIDs hypersensitivity. Fourteen patients (47 %) had a history of at least one previous relapse after FESS whereas 16/30 (53 %) were at their first surgery. Twenty-one patients (70 %) experienced relapse during their disease history (7/21 relapses occurred during the 27 ± 7 months period of prospective observation).

**Table 1:** clinical characteristics of the study population

Clinical parameter	N (%)
Males/females	19/11
Mean age (years)	52 ± 11.6
Family history for allergic diseases	11/30 (37 %)
Atopy (prick test and specific IgE to common aeroallergens)	18/30 (60 %)
New diagnosis	11/18 (61 %)
Asthma	16/30 (53 %)
New diagnosis	9/16 (56 %)
NSAIDs hypersensitivity	10/30 (33 %)
Relapsing forms	21/30 (70 %)
Blood hypereosinophilia before FESS	10/29 (34 %)

### Allergological parameters and association of atopy with relapse

**Fig. 1:**
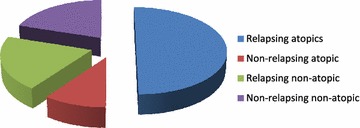
Frequency of atopy in relapsers: there was a trend of relapse in atopics versus non atopic subjects (*p* = 0.05).

**Fig. 2:**
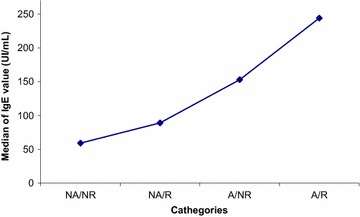
IgE levels considering atopy and relapse: higher levels of IgE were detected in atopics, in particular in relapsed ones.

The detailed description of allergycharacteristics of the 30 CRSWNP patients are shown in Additional file 1: Table S1. Eighteen patients (60 %) were atopic: 15 relapsers (83 %) and 3 non relapsers (*p* = 0.05, RR: 5, IC: 0.93–26.78) Fig. 1. Six out of 18 atopic patients (33 %) were monosensitized, whereas 12 (67 %) polysensitized. Three out of 18 (16.6 %) were sensitized to a seasonal allergen, whereas 15/18 (83.4 %) were sensitized also to a perennial allergen (house dust mites, moulds and/or pets dander). The median IgE concentration was higher in atopic, in particular in relapsers (Median: 161.5 UI/mL in relapsersversus 79 UI/mL in non-relapsers, ns), as showed in Fig. 2. Three subjects had high level of total IgE, without a sensitization to common aeroallergens. Two of them relapsed after surgery.

### Asthma

We found that 16/30 (53 %) patients were asthmatics. The diagnosis of asthma was made for the first time with us in 9 out of 16 asthmatic patients (56 %) of our group. Among asthmatics 13/16 (81 %) were relapsers (ns). The phenotype of CRSwNP patients with both asthma and atopy was more associated with relapsing forms of CRSwNP (*p* = 0.05).

### Markers of eosinophilic inflammation

**Fig. 3:**
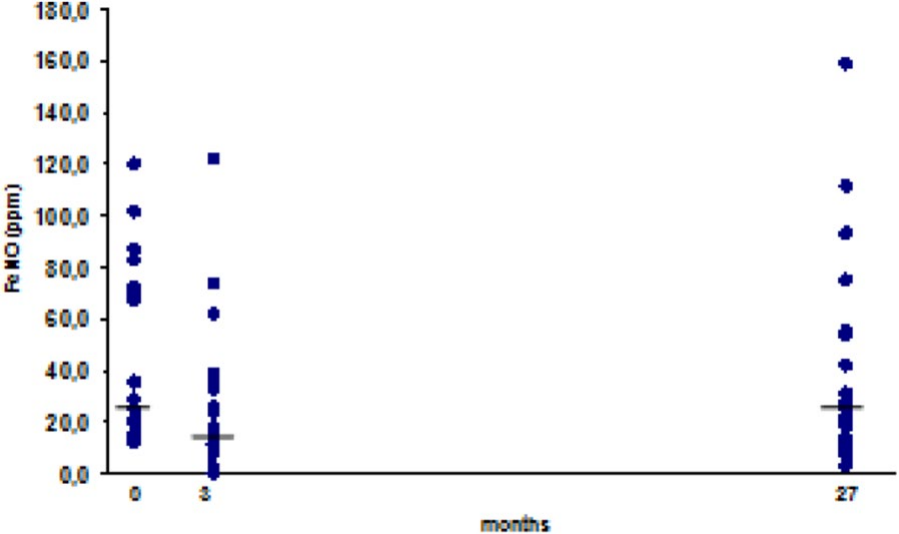
Timecourse of FeNO at baseline and at the short and long-term follow-up: the mean FeNO decreased after FESS (43.1–26.6 ppb) in 84 % of patients who performed the analysis both before and after FESS. However after 27 ± 7 months higher levels of FeNO were again detected (FeNO = 37.7 ppb at 27 months).

**Fig. 4:**
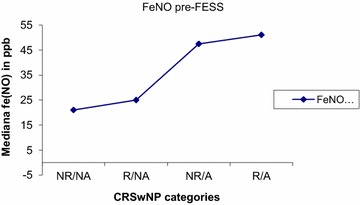
FeNO levels before surgery in atopic and non-atopic patients: higher levels of FeNO pre-FESS were detected in atopics, and in particular in relapsed ones.

**Fig. 5:**
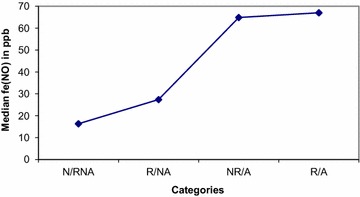
FeNO levels before surgery in asthmatic and non-asthmatic patients: higher levels of FeNO pre-FESS were detected in asthmatics, and in particular in relapsed ones.

Eight out of 21 relapsers (38 %) had increased blood eosinophilia versus 2/9 (22 %) non-relapsers (ns). Nitric oxide in exhaled air (FeNO) was evaluated both in asthmatics/non asthmatics and atopics/non atopics (Table 2). High levels of FeNO (>25 ppb) were detected in 50 % of patients at baseline and at the end of the follow-up. More than 50 % of patients had high levels of FeNO (>25 ppb) at baseline and at the end of the follow up. The mean FeNO decreased after FESS (43.1–26.6 ppb) in 84 % of patients who performed the analysis both before and after FESS (Fig. 3). However after 27 ± 7 months higher levels of FeNO were again detected (FeNO = 37.7 ppb at 27 months; Fig. 3). Higher levels of FeNO pre-FESS were detected in atopic (median: 30.10 vs 24.85 ppb in non atopic patients ns), and in particular in relapsed atopics versus non relapsed ones (median 51.1 ppb vs 22.1, ns). Higher levels of FeNO pre-FESS were detected inasthmatic patients (median FeNo levels 67 vs 19.2 ppb in non asthmatic patients; *p* = 0.05), especially in those who relapsed (median: 67 vs 64.85 ppb in non-relapsed patients, ns) (Figs. 4, 5).

**Table 2:** Percentage of patients with high FeNO (>25 ppb)

	Before FESS (n = 22/30)^a^	3 months (n = 26/30)^a^	27 ± 7 months (n = 26/30)^a^
All patients (N = 22)	54.5 %	31 %	54 %
Non-asthmatic	33 %	37.5 %	43 %
Non-atopic	42 %	37.5 %	36 %
Asthmatic	67 %	62.5	57 %
Atopic	58 %	62.5	64 %

^a^Total number of subjects who performed FeNO assessment at each time point.

### Respiratory function tests

The Tiffeneau Index (FEV1/FVC) was significantly lower in asthmatic relapsers than in non relapsers asthmatics (94.7 ± 11.1 versus 105 ± 5.9—*p* = 0.04). Also FEV 1 showed a worse trend in relapsers, when compared to non relapsers (3.07L, 96.7 % pred versus 3.7, 103 % pred, ns).

## Discussion

Identifying pathogenic factors involved in the development of NP and clinical markers of post-surgical relapse are still important unmet needs in the ENT/allergological practice. Here we described a cohort of patients with CRSwNP undergoing FESS and we evaluated clinical, laboratory and functional parameters during the course of the disease and in a short prospective period.

Persistent and symptomatic inflammation of the nasal mucosa and paranasal sinuses characterize CRSwNP as part of a complex pathophysiological continuum of eosinophilic inflammation involving both the upper and lower respiratory tract (United Airways Disease) [21]. NSAIDs hypersensitivity and asthma are also part of this pathological process and show strong epidemiological and pathogenic associations with CRSwNP [9, 22, 23]. In particular, the severity of asthma has been linked with the risk of relapse in patients with CRSwNP, while surgical treatment of NP ameliorated functional parameters in asthmatic patients [24].

Here, we showed that FeNO levels, which better correlate with the degree of eosinophilic inflammation at the level of the (lower) airways, were higher in asthmatic patients and relapsers. FeNO levels readily decreased after FESS in CRSwNP. Thus, in addition to previous reports describing an improvement in asthma symptoms and in peak expiratory flow rate [24], we provide an indirect evidence that immunological responses occurring at the level of the higher airways are required to sustain inflammation in the lower part of the respiratory tract, in accordance with the United Airways Disease hypothesis [21].

According to the literature, there is a weak association between atopy and the risk of NP development and relapse [9, 22, 23]. Consequently, allergy testing are not included in the flowchart of CRSWNP patients suggested by EPOS guidelines [1]. However, local derangement of the immune function towards aberrant IgE responses contributes to the maintenance of inflammation in CRSwNP [25]. Accordingly, anti-IgE therapeutic regimens showed efficacy in preventing NP relapses [26] and in the treatment of recalcitrant forms of NP associated with asthma [27, 28]. In this setting, the potential role of systemically deranged IgE responses (i.e. atopy) is still debated [25, 29–31]. In our small set of patients with CRSwNP, atopy shows a high prevalence, possibly due to a selection bias towards more severe forms of CRSwNP, frequently associated with comorbidities, in particular asthma, NSAIDs hypersensitivity and atopy, sometimes accompanied by clinically significant respiratory symptoms. We also observed an association between NP relapse and atopy, and this association was confirmed in patients with allergic asthma. In accordance to a prominent role of local IgE response in the pathogenesis of CRSwNP, circulating levels of IgE were higher in relapsing forms of CRSwNP.

Chronic rinonasal inflammation could be supported by an aberrant response against exogenous well known aeroallergens (house dust mites, fungi) [31] or develop as the result of superantigen (e.g. from *S*. *aureus*) stimulation [12]. Along with this line, we could hypothesize that specific immunotherapy (SIT) could prove beneficial in CRSWNP. However, few studies investigated the association between sensitization to aeroallergens and NPs, and even fewer tested the efficacy of SIT in the treatment of CRSWNP and prevention of relapse after FESS surgery. In particular the only large trial published to date [32] reported no benefit with the use of SIT with respect to NP relapse. However, that study was strongly limited by the short period of SIT administration.

## Conclusions

CRSWNP could be associated with atopy, clinical allergy, asthma and NSAIDs hypersensitivity. Patients with CRSwNP need therefore to be evaluated by mean of a multidisciplinary approach. Our data suggest that the phenotype of patients with both asthma and atopy is associated with relapsing forms of CRSwNP. Patients with CRSWNP show also signs of aberrant IgE responses and eosinophilic inflammation. Local inflammation of the upper respiratory tract seems to contribute to the maintenance of inflammation at the level of lower airways. On the other hand, local indices of eosinophilic inflammation and aberrant IgE response do not correlate with the corresponding systemic counterparts and are probably more accurate as markers of disease and maybe as predictors of relapse in CRSWNP. More studies are required to confirm the link between atopy allergic asthma and relapsing forms of CRSwNP.

## Additional files

Additional file 1:
**Table S1.** Allergy characterization of the study population.
