# Supplementary material for: Are atopy and eosinophilic bronchial inflammation associated with relapsing forms of chronic rhinosinusitis with nasal polyps?
Source: Clin Mol Allergy. 2015 Sep 11;13(1):23. doi: 10.1186/s12948-015-0026-8 (PMC4566488; doi:10.1186/s12948-015-0026-8)
Supplement: Additional file 1: — Table S1. Allergy characterization of the study population. [file 12948_2015_26_MOESM1_ESM.docx]

**Table S1: Allergy characterization of the study population**

| **N** | **Gender (1=M, 2=F)** | **Relapse** | **Age** | **Familiarity for atopy (0=no;1=yes)** | **Blood eosinophilia pre-FESS (0/1)** | **Positivity of skin prick test** | | | | **Mono poly-sensitization**  **Mono=1, Poly=2** | **Total IgE dosage** | **Positivity of specific IgE dosage** | | | | **NSAIDs hypersensitivity** |
| --- | --- | --- | --- | --- | --- | --- | --- | --- | --- | --- | --- | --- | --- | --- | --- | --- |
|  |  |  |  |  |  | **Pollen (0/1)** | **House dust mites (0/1)** | **moulds (0/1)** | **Pets allergens (0/1)** |  |  | **Pollen (0/1)** | **House dust mites (0/1)** | **moulds (0/1)** | **Pets allergens (0/1** |  |
| 1 | 1 | 0 | 49 | 0 | 1 | 0 | 0 | 0 | 0 | 0 | 99 | 0 | 0 | 0 | 0 | 0 |
| 2 | 1 | 0 | 74 | 0 | 0 | 0 | 0 | 0 | 0 | 0 | 29 | 0 | 0 | 0 | 0 | 0 |
| 3 | 2 | 1 | 65 | 1 | 0 | 0 | 0 | 0 | 0 | 0 | 24 | 0 | 0 | 0 | 0 | 0 |
| 4 | 1 | 1 | 29 | 1 | 0 | 1 | 1 | 1 | 1 | 2 | 244 | 1 | 1 | 1 | 1 | 1 |
| 5 | 2 | 1 | 73 | 0 | 1 | 0 | 0 | 0 | 1 | 1 | 41 | 0 | 0 | 0 | 0 | 1 |
| 6 | 2 | 1 | 56 | 0 | 0 | 1 | 0 | 0 | 0 | 1 | ND | ND | ND | ND | ND | 0 |
| 7 | 1 | 1 | 40 | 0 | 0 | 1 | 0 | 1 | 0 | 2 | 48 | 1 | 0 | 0 | 0 | 1 |
| 8 | 1 | 0 | 61 | 1 | 0 | 1 | 0 | 0 | 1 | 2 | 241 | 1 | 0 | 0 | 1 | 0 |
| 9 | 1 | 1 | 47 | 0 | 0 | 0 | 0 | 0 | 0 | 0 | 183 | 0 | 1 | 0 | 0 | 1 |
| 10 | 1 | 0 | 41 | 0 | 0 | 0 | 0 | 0 | 0 | 0 | ND | 0 | 0 | 0 | 0 | 0 |
| 11 | 2 | 1 | 68 | 0 | 1 | 1 | 0 | 0 | 0 | 1 | ND | 1 | 1 | 0 | 0 | 0 |
| 12 | 2 | 1 | 39 | 0 | 1 | 0 | 0 | 0 | 0 | 0 | ND | 0 | 0 | 0 | 0 | 1 |
| 13 | 1 | 1 | 38 | 1 | 0 | 0 | 1 | 0 | 0 | 1 | ND | ND | ND | ND | ND | 0 |
| 14 | 1 | 1 | 46 | 1 | 0 | 1 | 1 | 0 | 1 | 2 | 293 | ND | ND | ND | ND | 0 |
| 15 | 1 | 1 | 43 | 1 | 1 | 1 | 1 | 0 | 1 | 2 | 275 | 1 | 1 | 0 | 1 | 1 |
| 16 | 1 | 1 | 51 | 1 | 0 | 1 | 0 | 0 | 1 | 2 | ND | ND | ND | ND | ND | 0 |
| 17 | 2 | 0 | 47 | 0 | 0 | 0 | 0 | 0 | 0 | 0 | 13 | 0 | 0 | 0 | 0 | 1 |
| 18 | 2 | 1 | 50 | 1 | 0 | 1 | 1 | 0 | 0 | 2 | ND | ND | ND | ND | ND | 1 |
| 19 | 2 | 1 | 41 | 1 | 1 | 1 | 1 | 1 | 1 | 2 | 415 | 1 | 1 | 0 | 1 | 1 |
| 20 | 1 | 0 | 59 | 0 | 0 | 0 | 0 | 1 | 0 | 1 | 59 | 0 | 0 | 1 | 0 | 0 |
| 21 | 1 | 1 | 56 | 0 | 0 | 1 | 0 | 0 | 1 | 2 | 276 | 1 | 0 | 1 | 0 | 0 |
| 22 | 1 | 0 | 56 | 0 | 1 | 0 | 0 | 0 | 0 | 0 | 190 | 0 | 0 | 1 | 0 | 0 |
| 23 | 2 | 1 | 44 | 0 | 0 | 0 | 0 | 0 | 0 | 0 | 89 | 0 | 0 | 0 | 0 | 0 |
| 24 | 1 | 0 | 54 | 0 | 0 | 1 | 0 | 1 | 1 | 2 | 36 | 1 | 0 | 0 | 1 | 0 |
| 25 | 1 | 1 | 66 | 0 | 1 | 0 | 1 | 1 | 0 | 2 | 127 | 0 | 1 | 0 | 0 | 0 |
| 26 | 1 | 1 | 58 | 0 | 1 | 0 | 0 | 0 | 0 | 0 | 140 | 0 | 0 | 0 | 0 | 0 |
| 27 | 2 | 1 | 64 | 0 | 1 | 0 | 0 | 0 | 0 | 0 | 34 | ND | ND | ND | ND | 1 |
| 28 | 2 | 0 | 32 | 1 | 0 | 1 | 1 | 0 | 0 | 2 | 153 | 1 | 1 | 0 | 0 | 0 |
| 29 | 1 | 1 | 50 | 0 | 0 | 1 | 0 | 0 | 0 | 1 | ND | ND | ND | ND | ND | 0 |
| 30 | 1 | 1 | 63 | 0 | 0 | 0 | 0 | 0 | 0 | 0 | 1051 | 0 | 0 | 0 | 0 | 0 |
